# Supplementary material for: Inhibition of CXCR4 Enhances the Efficacy of Radiotherapy in Metastatic Prostate Cancer Models
Source: Cancers (Basel). 2023 Feb 6;15(4):1021. doi: 10.3390/cancers15041021 (PMC9954510; doi:10.3390/cancers15041021)
Supplement: Supplementary file 1 [file cancers-15-01021-s001.zip › cancers-1901907-supplementary.pdf]

Supplementary Table S1. Antibodies and dilutions used for IHC in this study.

Antibody	Company	Catalog No.	Dilution
Western Blot			
vimentin	Cell Signaling	5741	1:1000
snail	Cell Signaling	3879	1:1000
slug	Cell Signaling	9585	1:1000
ZEB1	Abcam	ab124512	2 μg/ml
NG2	Millipore	AB5320	1:1000
CXCR4	Abcam	ab2074	2 μg/ml
CXCR7	Abcam	ab72100	2 μg/ml
desmin	R&D Systems	AF3844	10 μg/mL
GAPDH	Cell Signaling	5174	1:1000
β-actin	Sigma Life Science	A 5316	1:5,000
rabbit HRP	Cell Signaling	7074	1:1000
mouse HRP	GE Healthcare	ab6728	1:5000
IHC/IF			
CD31	Millipore	MAB1398Z	1:2000
NG2	Millipore	AB5320	1:250
CXCR4	Abcam	ab2074	1:100
CXCR7	Abcam	ab72100	1:100
SDF1α	Cell Sciences	CPS000	1:500
CA-IX	Abcam	ab15086	1:500
Secondary antibodies for immunofluorescence (Cy3, Cy5)	Jackson Immunoresearch	N/A	1:200
Secondary antibodies for IHC (ABC-DAB staining kits)	Vector Laboratories	SK-4105	N/A

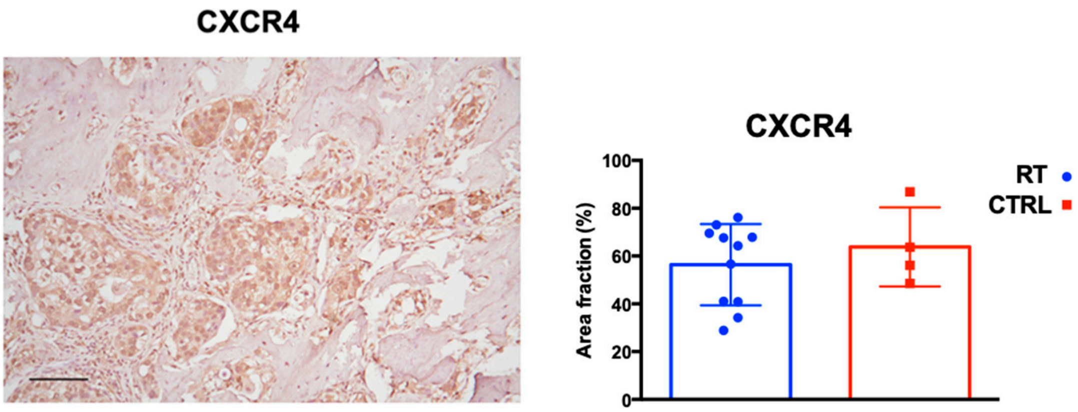

Figure S1: Expression of CXCR4 in human bone metastatic prostate cancer tissues. Left, representative IHC; right, quantification of CXCR4 staining.

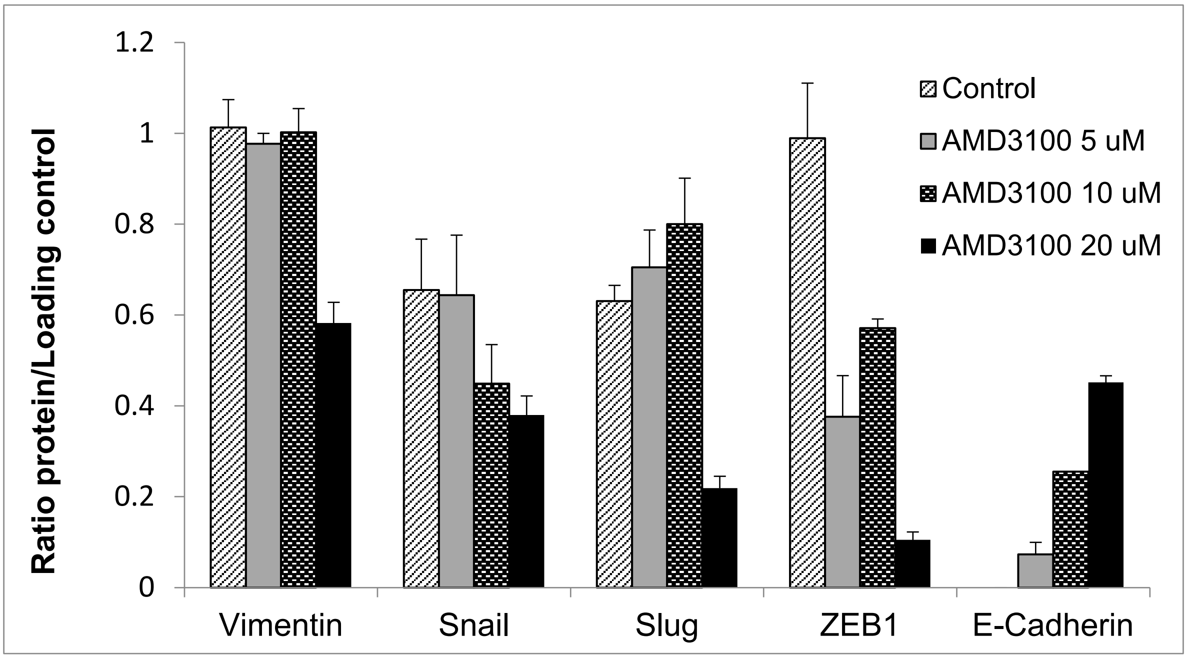

Figure S2: Densitometry for Western blot data in Figure 2b.

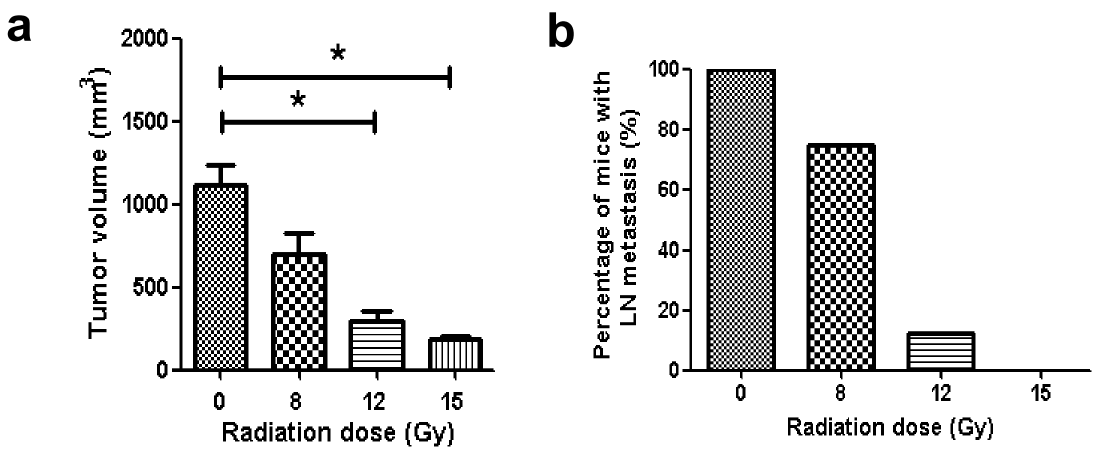

Figure S3: Radiation dose-dependent inhibition of tumor growth in C4-2B PCa xenografts in the prostate. (**a**) Primary tumor growth inhibition. (**b**) Effect on lymph node (LN) metastasis.

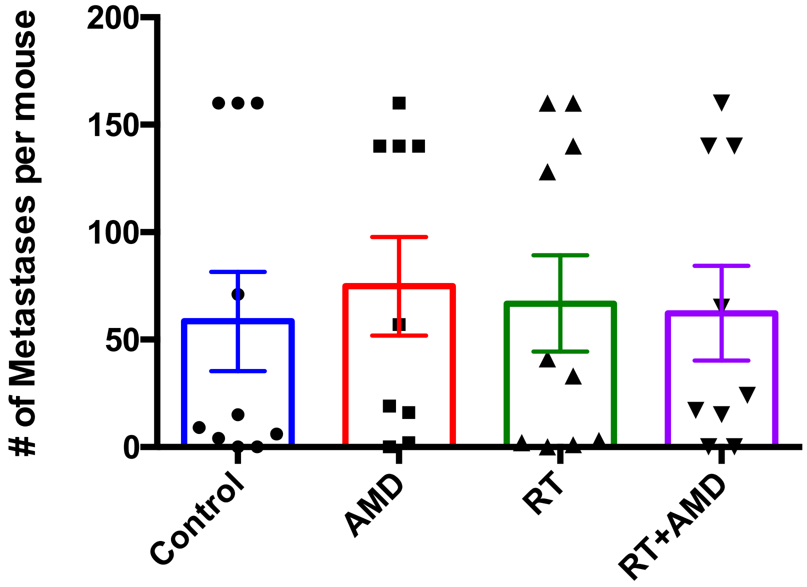

Figure S4: Effect of radiotherapy with AMD3100 treatment on secondary lung metastasis at the terminal endpoint in the *PtenSmad4-null* mPCa model.

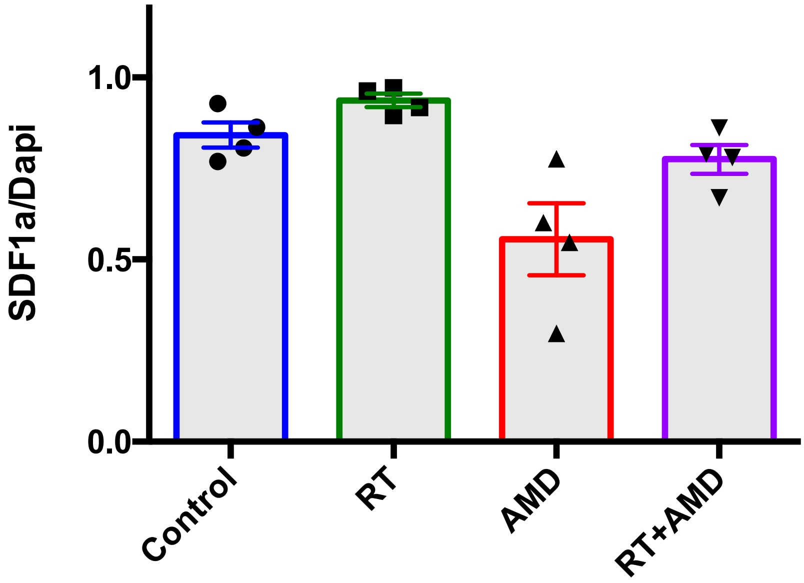

Figure S5: Changes in SDF1α expression in *PtenSmad4*-null metastatic prostate cancer following radiotherapy, AMD3100 treatment or their combination versus control.

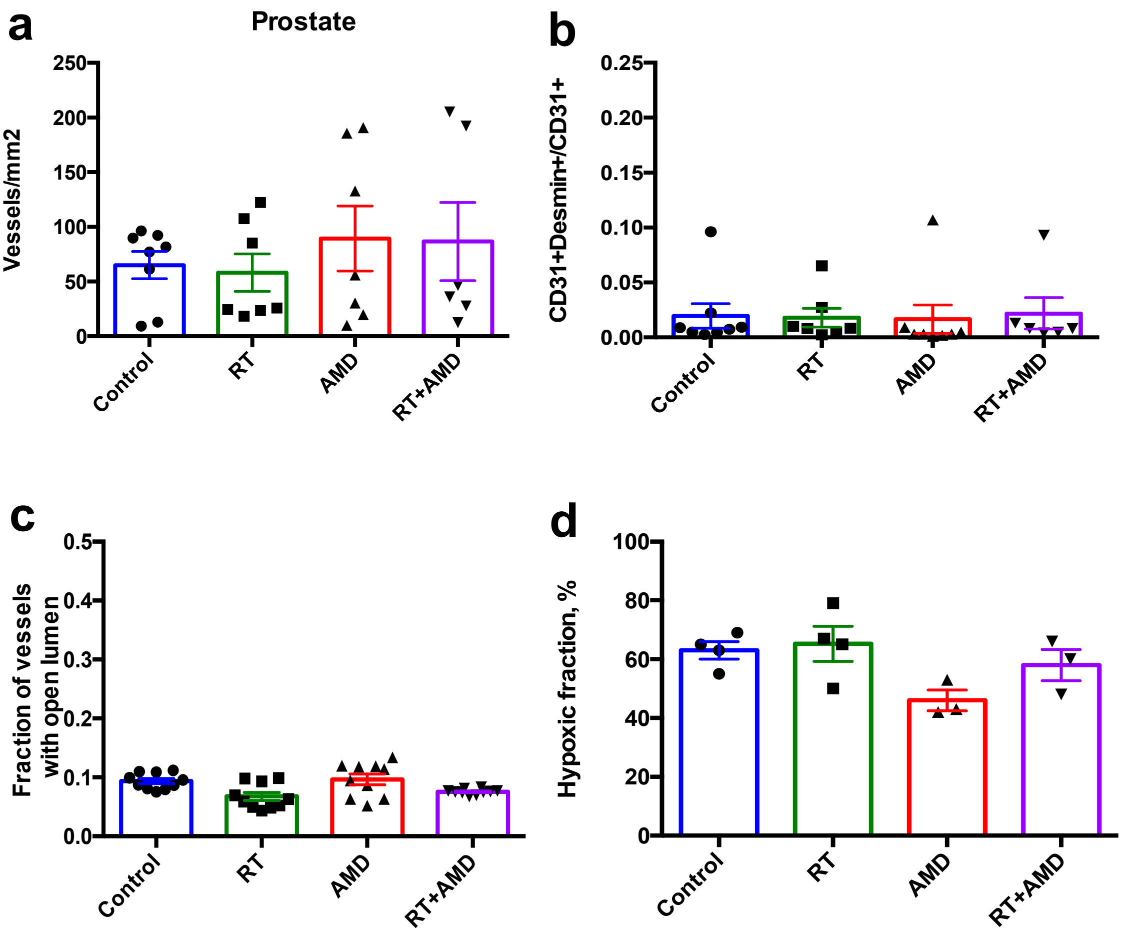

Figure S6: Changes in tumor vascular parameters induced by radiotherapy, AMD3100 treatment or their combination versus control in *PtenSmad4*-null prostate cancer implanted in the prostate. (**a**) Vascular density. (**b**) Mature vessel fraction. (**c**) Vessels with open lumens. (**d**) Hypoxic tissue fraction.

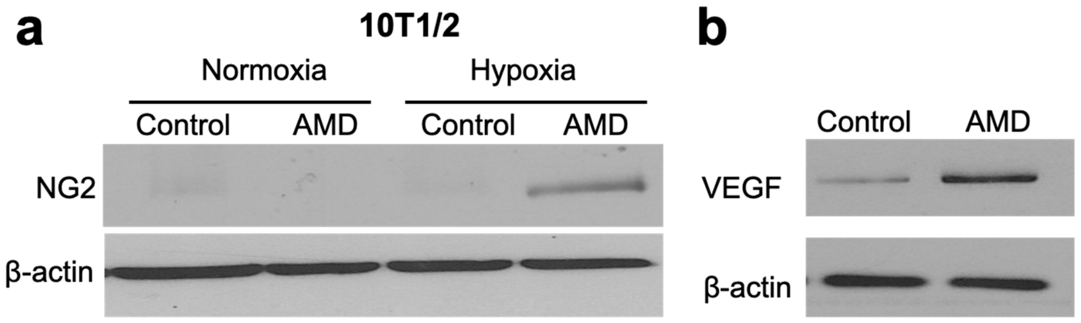

Figure S7: Effects of CXCR4 inhibition using AMD3100 on 10T1/2 cells perivascular precursor cells. (**a**) NG2 expression in normoxic and hypoxic conditions. (**b**) VEGF-A expression in hypoxic conditions.

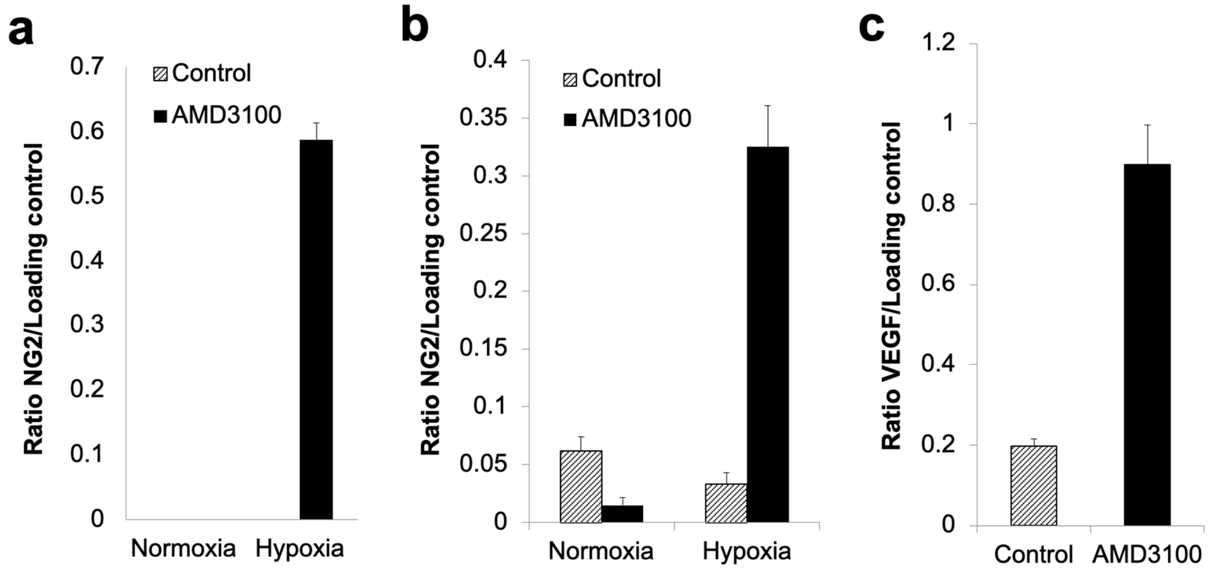

Figure S8: Densitometry for Western blot data in Figure 6a (a), Figure S7a (b), and Figure S7b (c).
